# Impact of tourism activities on the distribution and pollution of soil heavy metals in natural scenic spots on the northern slope of Tianshan Mountain

**DOI:** 10.1371/journal.pone.0267829

**Published:** 2022-07-27

**Authors:** Jianjun Yang, Huan Xu, Xinjun Wang

**Affiliations:** 1 College of Resource and Environment Sciences, Xinjiang University, Urumqi, China; 2 Key Laboratory of Oasis Ecology, Ministry of Education, Urumqi, China; 3 College of Grass Industry and Environment, Xinjiang Agricultural University, Urumqi, China; Ghazi University, PAKISTAN

## Abstract

Human activities can significantly impact the natural ecosystem. As an important part of terrestrial ecosystems, soil participates in energy and material cycle. With the continuous intensification of human activities, soil undergoes increasingly serious disturbance. Under the influence of global change and human activities, the variability of heavy metals in soil is worthy of further discussion. Taking Lujiaowan and Juhuatai Scenic Spot on the northern slope of Tianshan Mountain as the research area, this study investigated the concentrations of Pb, As, Zn, Cu and Mn in the soil and analyzed their distribution and pollution levels. Meanwhile, the main sources of soil heavy metals were explored using autocorrelation analysis and principal component analysis. Results showed that the order of the average concentration of heavy metals in the study area was as follows: Mn> Zn> Cu> Pb> As. None of them exceeded the national soil environmental quality level II standard, and the average concentrations of Zn, Cu, and As exceeded the background value of Xinjiang soil, reaching a light pollution level. In addition, the distribution of heavy metals in soil displayed a regular trend, and a positive correlation was found between disturbance intensity and heavy metal concentration. The geoaccumulation index also showed that the five heavy metals in the study area had lower pollution degree compared with the background value in Xinjiang. The order of potential ecological risk was As > Cu > Pb > Zn > Mn.

## 1. Introduction

As an important natural resource, soil plays an important role in absorbing and purifying pollutants in the environment. Soil pollution occurs when the pollutant concentration in soil exceeds a specific value, consequently influencing soil structure, function, and plant growth [1–3]. Ecosystem degradation may occur if soil pollutants exceed the purification capacity of soil [4]. Among various soil pollutants, heavy metals pollution, which refers to environmental pollution caused by heavy metals or their compounds, and mainly caused by mining, waste gas discharge, sewage irrigation and the use of products with excessive heavy metals, are characterized by bioaccumulation, toxicity, and persistence and could cause extremely negative impacts and is relatively difficult to treat [5]. Heavy metals enter the human body through food chain enrichment and affect human health by disrupting the circulation, nervous, digestive, endocrine, and reproductive systems [6–9]. Currently, a lot of research on soil heavy metal pollution concentrated on industrial and mining areas, farmland, and other areas where human activities are intensive [1, 2, 10–13], and few studies focused on nature reserves and scenic spots. The transportation and tourism in scenic spots may result in accumulation of heavy metals in the soil, affecting the soil ecosystem around the scenic spots. Therefore, exploring the distribution and pollution level of heavy metals in the soil of scenic spots has great theoretical and practical significance [14–16].

Existing research shows that tourism activities significantly affect the concentration of heavy metals in the soil near scenic spots, and most studies believe that tourism activities increase the concentrations of some heavy metals in soil [14, 17, 18]. The heavy metal pollution of tourist scenic spots caused by human activities and their potential ecological risks vary in different regions [14, 19]. Shi et al used the Nemero index method to evaluate the soil heavy metal pollution of parks in Shanghai and found that the serious heavy metal pollution in Shanghai urban parks may come from traffic and industrial pollution, which has no significant correlation with the completion time of the park [20]. Another study in the Songshan scenic area of China showed that the concentrations of heavy metals (Cu, Zn, Pb, Ni, Cd, and Cr) in the soil caused by tourism exceed the background value, and the pollution degree is ranked as follows: Zn > Pb > Cd > Cr (No Cu and Ni contamination was observed) [21]. Assessment of heavy metal pollution risks and enzyme activity of meadow soils in urban area under tourism load conducted in Poland found that soils of tourist areas are under stronger negative impact than soils of the town center because of the cumulative effect of transport of heavy metals from the city center, pollution by skiing machinery, and melting water from the artificial snow [15].

Actually, the concentration of soil heavy metal might be correlated to the intensity of human disturbances, which indicates human pressure on the landscape [22]. Compared with other regions, in arid areas with fragile ecological environment, uneven temporal and spatial distribution of water resources and limited ecosystem service functions, the disturbance of human activities on natural ecosystems may lead to more serious consequences. We can speculate that since the disturbance of human activities on scenic spots located in arid areas is stronger than other non-scenic areas, the concentration of heavy metals in soil of scenic spots may be higher. Unfortunately, the current researches have not conducted an in-depth study on this (different intensities of human activities may lead to different contents of heavy metals in the soil of scenic spots located in arid areas), which restricted our understanding on the mechanism of the response of soil to human disturbances in arid regions.

Taking the northern slope of Tianshan Mountain as the research object, the present study selected two typical tourist areas (Lujiaowan and Juhuatai) to analyze the distribution and pollution degree of soil heavy metals under different tourism disturbance intensities and to explore the main sources of soil heavy metals. Combined with the geoaccumulation index method and potential ecological risk index method, the potential ecological risk level of soil heavy metals in the study area was evaluated. The corresponding results could provide reference for the environmental protection of the arid regions in Central Asia.

## 2. Materials and methods

### 2.1 Study area

Lujiaowan scenic area is located between 84°56′–85°26′E and 43°49′–44°04′N, with an altitude of more than 4000 m. The average temperature in summer is 20°C, the average temperature in winter is -14°C, and the annual rainfall is more than 600 mm. Juhuatai scenic spot is located at 87°10′–87°15′E, 43°29′–43°47′N, with an altitude of 2000–2400m, an average annual temperature of 2°C–4°C, a frost-free period of 130–150 days, and an annual precipitation of 270–300 mm. The vegetation of these two spots mainly includes coniferous forest and grassland. The soil is mainly composed of Mountain grey cinnamon soil.

### 2.2 Soil sampling and laboratory analysis

Soil samples were collected in May 2019. Sampling information is presented in Tables 1 and 2. According to the land cover and disturbance of the study area, four sampling areas including severe disturbance, moderate disturbance, no disturbance and hiking trail were set up in the above mentioned two scenic spot (Lujiaowan and Juhuatai). During fields investigations, the geographical coordinates, vegetation types and soil type of the sampling area were recorded. In each of the scenic spot (Lujiaowan and Juhuatai), three sampling centers were randomly selected in sampling area of severe disturbance, moderate disturbance, and hiking trail, for the sampling area of no disturbance, only one sampling center were selected. Therefore, ten sampling centers were selected at each of the scenic spot (Lujiaowan and Juhuatai). Around each of the sampling center, five sampling plots (1m×1m square) were set at 2.5m, 5m, 10m, 15m and 20m away from the sampling center and soil samples were collected at 0–10 cm and 10–20 cm depths. As a result, a total of 200 soil samples were collected and brought back to the laboratory. During laboratory analysis, the plant residues were removed, the soil bulk density and water content were measured. Then, the soil samples were air-dried and sieved (10 mesh). The concentration of heavy metals was determined using portable X-ray spectrometer, which has been proved to be a suitable method [22–24].

**Table 1:** Overview of sampling points.

Study area	Sampling area	Coordinates	Soil type	Vegetation type	bulk density(g/cm^3^)	soil moisture content(%)
Lujiaowan	No disturbance area	43°96′N, 85°15′E	Chernozem	Grassland	0.76±0.05	0.24±0.03
	Moderate disturbance area	43°98′N, 85°16′E	Chernozem	Grassland	1.12±0.02	0.23±0.01
	Severe disturbance area	43°97′N, 85°11′E	Chernozem	Grassland	1.08±0.02	0.12±0.01
	Hiking trail area	43°97′N, 85°11′E	Grey cinnamon soil	Grassland	0.96±0.02	0.21±0.01
Juhuatai	No disturbance area	43°45′N, 87°13′E	Grey cinnamon soil	Grassland	0.98±0.08	0.23±0.02
	Moderate disturbance area	43°45′N, 87°11′E	Alpine meadow soil	Forest	1.19±0.05	0.22±0.01
	Severe disturbance area	43°45′N, 87°12′E	Alpine meadow soil	Grassland	0.95±0.03	0.16±0.01
	Hiking trail area	43°46′N, 87°12′E	Grey cinnamon soil	Grassland	0.98±0.03	0.21±0.01

**Table 2:** Soil particle size composition.

Study area	Sampling area	1.0–2.0mm	0.5–1.0mm	0.25–0.5mm	0.1–0.25mm	<0.1mm
Lujiaowan	No disturbance area	4.90±0.93	17.64±2.09	25.12±1.44	20.06±0.95	32.28±2.6
	Moderate disturbance area	1.43±0.35	7.31±1.19	19.12±0.80	26.82±0.85	45.32±1.47
	Severe disturbance area	6.32±0.73	21.52±1.08	22.64±0.71	20.33±0.53	29.19±1.12
	Hiking trail area	4.44±0.45	18.82±1.15	24.46±0.97	23.09±0.65	29.19±1.52
Juhuatai	No disturbance area	5.29±1.25	15.80±2.97	21.66±0.72	23.83±1.82	33.43±2.55
	Moderate disturbance area	6.47±0.92	20.55±1.91	21.88±0.78	20.91±0.87	30.19±2.24
	Severe disturbance area	4.89±0.74	23.69±1.49	23.15±0.64	21.25±0.65	27.02±1.64
	Hiking trail area	5.59±0.60	21.93±1.42	23.58±0.54	20.06±0.63	28.84±1.24

### 2.3 Statistical analyses

The pollution index (single factor) is widely used in evaluating the heavy metal pollution in various environments [23–26]. If define *Pi* is the single factor index of metal *i* in soil, then *P*_*i*_ *= C*_*i*_*/S*_*i*_, where *C*_*i*_ represents the measured concentration of metal *i* in soil and *S*_*i*_ is geochemical background value as mentioned previously. According to the value of *P*_*i*_, the environmental pollution could be categories to four classes as shows in Table 3.

**Table 3:** Single factor pollution index criteria.

Range of P_i_	Pollution level
P_i_≤1	Uncontaminated
1<P_i_≤2	Light contaminated
2<P_i_≤3	Moderate contaminated
3<P_i_	Heavily contaminated

In the present study, the geoaccumulation index (*I*_*geo*_) was used to evaluate the heavy metal pollution in our study area. The *I*_*geo*_ was defined by Müller [27] and expressed as *I*_*geo*_ *= log*_*2*_ *(Cn/1*.*5 × B*_*n*_*)*, where *Cn* is the concentration of heavy metals in soil and *Bn* is the geochemical background value. According to Müller [27] and the actual pollution in our study area, we defined six classes of *I*_*geo*_ (Table 4), ranging from class 0 (*I*_*geo*_ = 0, means unpolluted) to class 5 (*I*_*geo*_ > 4, denotes extremely polluted).

**Table 4:** Geoaccumulation Index classes.

Class	I_geo_ value	Soil pollution category
0	I_geo_ ≤0	Uncontaminated
1	0 < I_geo_ ≤ 1	Uncontaminated to moderately contaminated
2	1 < I_geo_ ≤ 2	Moderately contaminated
3	2 < I_geo_ ≤ 3	Moderately to heavily contaminated
4	3 < I_geo_ ≤ 4	Heavily contaminated
5	4 < I_geo_	Extremely contaminated

As an important indicator used to assess the environmental risk induced by concentration of heavy metals in soil, the potential ecological risk (RI) has been widely used since it was introduced [28]. The RI is defined as $RI=\sum E_{r}^{i}=T_{r}^{i}\times P_{i}$, where $E_{r}^{i}$, m, $T_{r}^{i}$, and *P*_*i*_ are ecological risk factor, number of heavy metals, toxicity response coefficient and *P*_*i*_ single pollution index of heavy metals, respectively. In the present study, we calculated the RI use $T_{r}^{i}$ and then defined five environmental risk classes as shown in Table 5.

**Table 5:** Potential ecological risk index criteria and toxicity response coefficient of heavy metals.

${\text{E}}_{\text{r}}^{\text{i}}$	Ecological risk level	RI	Ecological risk level	heavy metal	Background value of Xinjiang	Corresponding coefficients
${\text{E}}_{\text{r}}^{\text{i}}$≤40	Low	RI<150	Low	Pb	19.40	5
40<${\text{E}}_{\text{r}}^{\text{i}}$≤80	Moderate	150<RI≤300	Moderate	As	11.20	10
80<${\text{E}}_{\text{r}}^{\text{i}}$≤160	Higher	300<RI≤600	Higher	Zn	68.80	1
160<${\text{E}}_{\text{r}}^{\text{i}}$≤320	High	600<RI≤1200	High	Cu	26.70	5
320<${\text{E}}_{\text{r}}^{\text{i}}$	Serious	RI>1200	Serious	Mn	688	1

Since it was proved that two heavy metals with a significant correlation coefficient may share the same origin, then correlation analysis was used to identify the origin of elements. PCA is a statistical method that transforms multiple variables into a few principal components. It is an important dimension reduction method that can reduce the dimension of high-dimensional data and the number of predictive variables [29, 30]. In this study, PCA was used to infer the main sources of soil heavy metals.

## 3. Results

### 3.1 Horizontal distribution of heavy metals in soil

**Fig 1:**
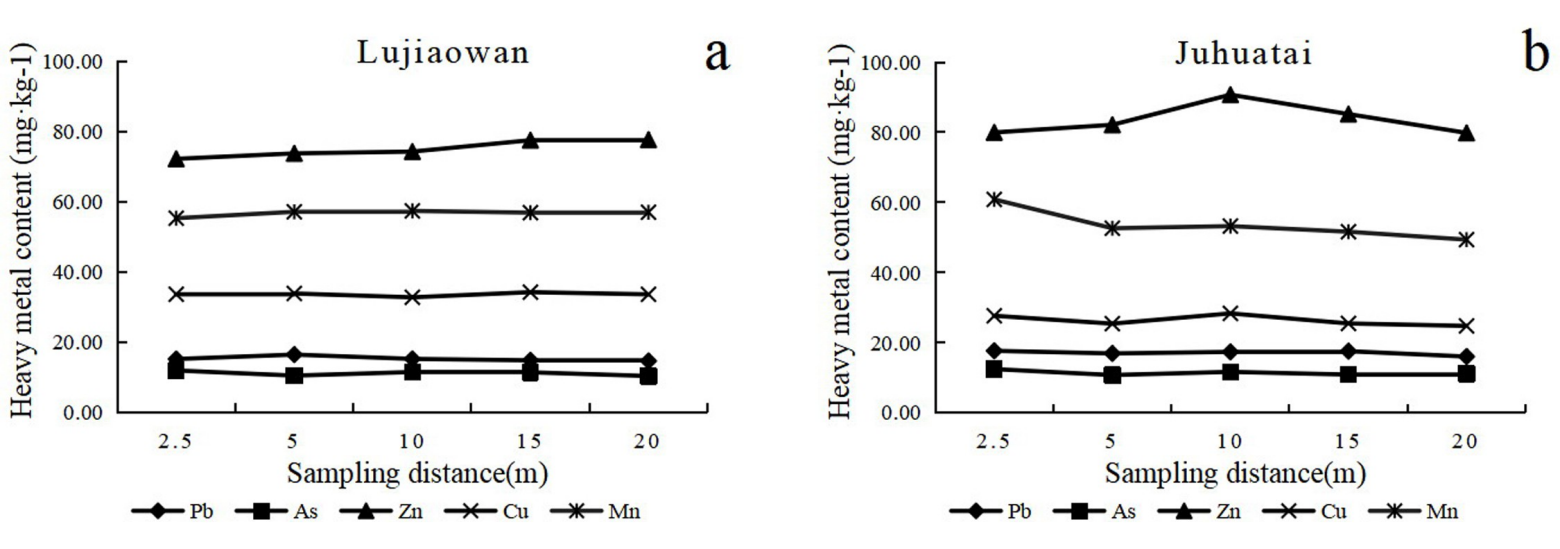
Concentrations of soil heavy metal at different sampling plots.

**Fig 2:**
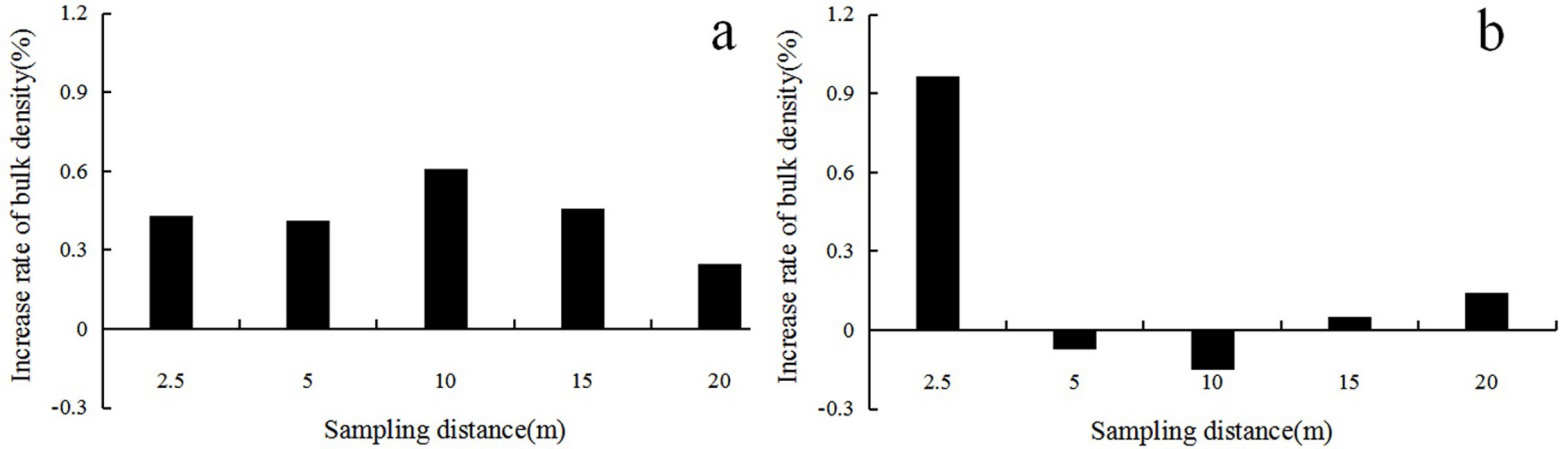
Soil bulk density at different sampling plots.

The horizontal distribution of soil heavy metal concentration is shown in Fig 1, and the increase rate of soil bulk density is demonstrated in Fig 2 (where a value less than 0.2 means slight trample, 0.2–0.3 means moderate trample, and more than 0.3 means severe trample). The maximum concentrations of As and Pb in Lujiaowan scenic spot appeared at 2.5 and 5 m away from the sampling centers and decreased with the increase in distance. The variation of soil bulk density (Fig 2) reflected the impact of human activities. The maximum concentration of Zn and Mn appeared at 20 m from the sampling centers, and the concentration increased with increasing distance. Compared with those at the closer sampling sites, the bulk density was higher at 20 m, and the heavy metals on the soil surface were not easy to migrate, indicating that human activities significantly affected soil physical and chemical properties.

At Juhuatai, the maximum concentrations of soil Pb, As, and Mn appeared at 2.5 m away from sampling centers and decreased with increasing distance (Fig 1B). Among the four heavy metals, the concentration of Mn in soil decreased the greatest. With the increase in sampling distance, the concentration of Zn in soil demonstrated an increasing–decreasing trend, and the maximum value appeared at 10 m. With the increase in sampling distance, the concentrations of different heavy metals showed different characteristics, and similar results can be found in other regions [31–33]. Even in a small space, the response of each element to soil physical and chemical properties and human activities may be different [34, 35].

### 3.2 Vertical distribution of heavy metals in soil

The average concentration of Pb, As, Zn, Cu, Mn in 0–10cm soil of Lujiaowan are 15.73 ± 3.02 mg·kg^-1^, 11.18 ± 2.84 mg·kg^-1^, 77.53 ± 11.08 mg·kg^-1^, 31.93 ± 7.87 mg·kg^-1^ and 560.93 ± 88.72 mg·kg^-1^, respectively. In 10–20 cm depth, the average concentration of Pb, As, Zn, Cu, Mn are 14.78 ± 4.43 mg·kg^-1^, 11.05 ± 2.37 mg·kg^-1^, 72.57 ± 9.61 mg·kg^-1^, 35.28 ± 8.55 mg·kg^-1^ and 573.30 ± 87.94 mg·kg^-1^, respectively. At Juhuatai, the average concentration of Pb, As, Zn, Cu, Mn in 0–10cm soil are 17.32 ± 3.83 mg·kg^-1^, 11.08 ± 2.69 mg·kg^-1^, 84.87 ± 18.27 mg·kg^-1^, 27.84 ± 6.03 mg·kg^-1^ and 546.52 ± 121.46 mg·kg^-1^, respectively. In 10–20 cm depth, the average concentrations are 16.81 ± 2.72 mg·kg^-1^, 11.58 ± 2.38 mg·kg^-1^, 82.25 ± 21.04 mg·kg^-1^, 24.75 ± 4.29 mg·kg^-1^ and 523.98 ± 99.30 mg·kg^-1^, respectively. It can be noted that not only the average concentrations of heavy metal in 10–20 cm soil are relatively lower, the variations (as denoted by standard deviation) of concentrations are also lower in the 10–20 cm soil, compare with 0–10 cm soils. The relatively low variation of concentration in deeper soil are in line with previous studies [36–38], since the deeper soil are less disturbed by human activities.

**Fig 3:**
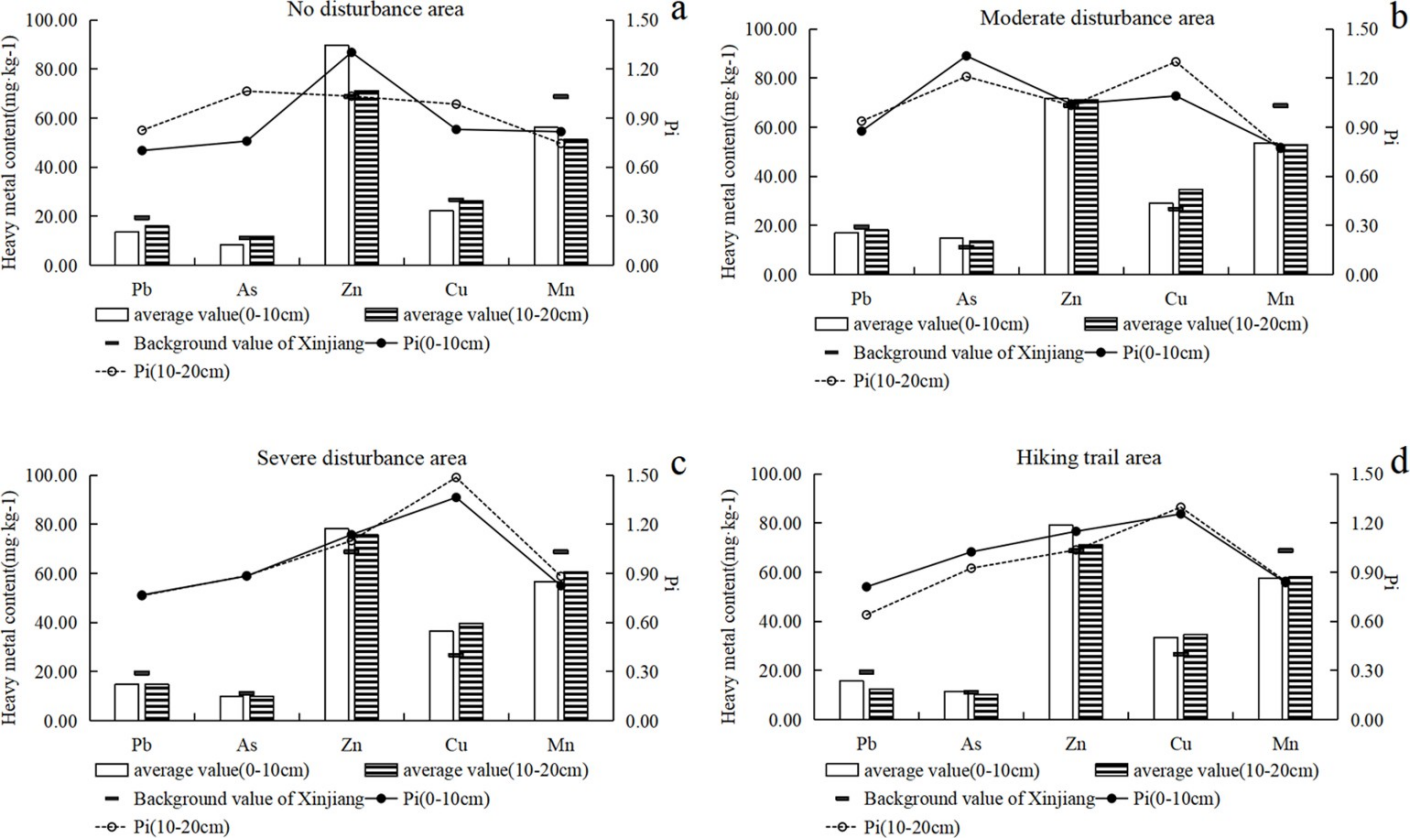
Heavy metal concentration in soil under different disturbance levels in Lujiaowan Scenic Area.

In the 0–10 and 10–20 cm depth soils of Lujiaowan, the Pi values of Zn were 1.30 and 1.03 in the soil of the no-disturbance area, 1.04 and 1.03 under moderate disturbance, 1.14 and 1.10 under severe disturbance, and 1.15 and 1.04 in the soil near the hiking trail, respectively (Fig 3). The concentration of Zn in the upper soil was higher than that in the lower soil, suggesting that natural factors and human activities jointly contributed to the surface concentration of heavy metals. In this area, the pollution index values of Cu in the 0–10 and 10–20 cm depth soils were 1.09 and 1.30 under moderate disturbance, 1.36 and 1.49 under severe disturbance, and 1.26 and 1.30 near the hiking trail, respectively. The higher the intensity of human activities, the higher the concentration of heavy metals in soil. In addition, the increase rate of heavy metal concentration in the deeper soil was higher than that in the shallower soil, indicating the surface accumulation of heavy metals.

**Fig 4:**
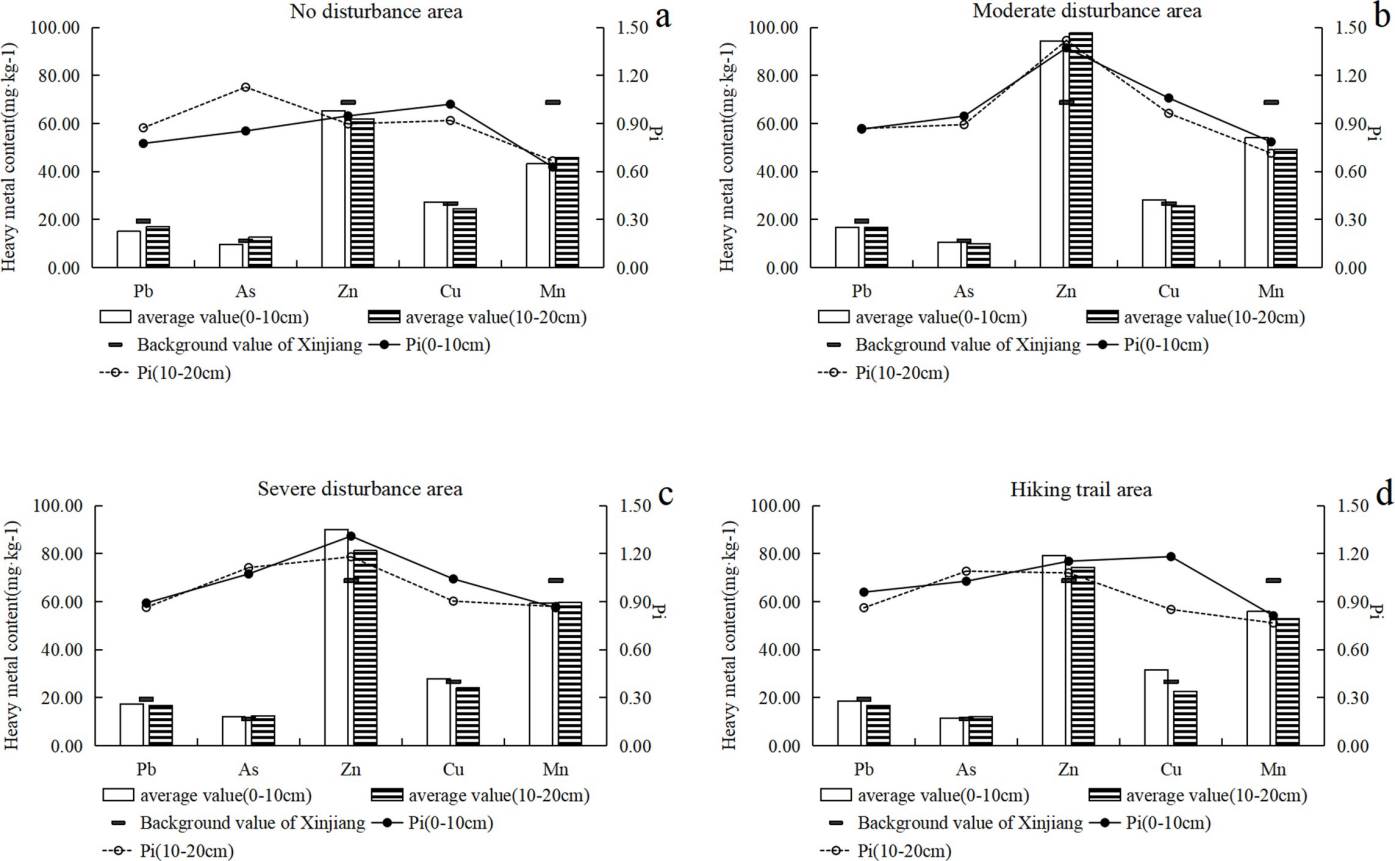
Heavy metal concentration in soil under different disturbance levels in Juhuatai Scenic Area.

In the 0–10 and 10–20 cm soil layers of Juhuatai scenic spot, the Pi values of As were 0.85 and 1.13 in the soil of no disturbance, 1.07 and 1.11 under severe disturbance, and 1.03 and 1.09 near the hiking trail, respectively (Fig 4). The concentration of deeper soil was higher than that of shallower soil, and the concentration of metal with heavy disturbance was higher than that of moderate disturbance. In Juhuatai, the accumulation of heavy metals increased in the deep layers.

### 3.3 Soil heavy metals corresponding to different disturbance level

The order of average concentration of heavy metals in the soil of the study area was Mn > Zn > Cu > Pb > As (Table 6). In the 0-10cm layer of Lujiaowan and Juhuatai scenic spots, the average concentrations of As (10.97 mg·kg^-1^, 11.37 mg·kg^-1^), Zn (75.95 mg·kg^-1^, 80.40 mg·kg^-1^) and Cu (32.05 mg·kg^-1^, 25.84 mg·kg^-1^) were higher than the background values of Xinjiang soil, reaching the level of light pollution. Generally, the concentrations of heavy metal were higher in moderate disturbance, severe disturbance and hiking trial area compare with no disturbance area, as shows in Table 6. The coefficient of variation of the five soil heavy metals in the study area was lower than 30%, indicating there is little difference among sampling sites. In addition, we found that the average concentration of heavy metals in soil was inversely correlated to the content of soil water. This is reasonable because the movement of soil water can cause the migration of heavy metal, as proved in experimental study conducted in Karst water system of Southwest China [39].

**Table 6:** Heavy metal concentration in soils of study area.

Study area	Disturbance level	Pb (mg·kg^-1^)	As (mg·kg^-1^)	Zn (mg·kg^-1^)	Cu (mg·kg^-1^)	Mn (mg·kg^-1^)
Lujiaowan	No disturbance area	14.83±0.98	10.22±0.81	80.41±4.27	24.26±1.66	537.78±26.27
	Moderate disturbance area	17.71±0.54	12.81±0.52	71.17±1.78	31.70±1.39	534.65±13.74
	Severe disturbance area	14.10±0.71	9.92±0.29	76.98±1.66	38.14±1.22	587.69±17.34
	Hiking trail area	14.08±0.66	10.92±0.44	75.23±2.01	34.09±1.54	588.78±15.67
Juhuatai	No disturbance area	16.00±0.75	11.10±1.00	63.51±2.25	23.48±1.93	445.85±33.08
	Moderate disturbance area	16.83±0.50	10.25±0.37	95.56±4.09	26.69±0.93	516.31±22.26
	Severe disturbance area	17.04±0.88	12.25±0.53	85.69±3.48	26.49±1.30	595.93±24.41
	Hiking trail area	17.68±0.67	11.88±0.48	76.83±2.39	26.71±0.96	543.54±14.05
Background value of Xinjiang		19.40	11.20	68.80	26.70	688.00
National soil environmental quality class II		350	25	300	100	—
Coefficient of Variation at Lujiaowan		0.23	0.18	0.13	0.20	0.16
Coefficient of Variation at Juhuatai		0.17	0.21	0.17	0.18	0.19

### 3.4 Source analysis of heavy metals in soil

The correlation coefficients between Pb and As and between Mn and As were 0.79 and -0.77 (p < 0.05), respectively (Table 7). Therefore, Pb and As and Mn and As have the same origin. As and Pb in soils may be affected by the entry of domestic waste from tourists, such as plastic products, glass bottles, and automobile exhaust, resulting in the enrichment of these heavy metals. In Juhuatai scenic spot, the correlation between the five heavy metals was low, indicating that their sources may be different. A previous study that investigated the heavy metal loads in forest soil close to a steel mill near Leoben, Austria similarly demonstrated that the close link between Pb and Zn suggests the same origin of these two metals [40]. Similar results were reported elsewhere [41–44], indicating that the origin of these heavy metals might be the garbage discarded by local residents and tourists.

**Table 7:** Correlation analysis of soil heavy metals.

Lujiaowan						Juhuatai				
	Pb	As	Zn	Cu	Mn	Pb	As	Zn	Cu	Mn
Pb	1					1				
As	0.79*	1				0.47	1			
Zn	-0.37	-0.65	1			0.31	-0.27	1		
Cu	-0.01	-0.08	-0.42	1		0.45	-0.36	0.26	1	
Mn	-0.69	-0.77*	0.60	-0.69	1	0.60	0.51	0.53	0.16	1

* statistically significant at p<0.05

The PCA revealed that the contributions of the first two principal components were 59.12% and 26.40%, respectively, and the cumulative variance was 85.52% at Lujiaowan (Table 8). Among these metals, Pb and As had relatively higher loadings on PC1, indicating that the variability of PC1 was controlled by Pb and As. Zn and Cu had relatively higher loadings on PC2. At Juhuatai, the variances of the first and second PCs were 45.13% and 31.52%, respectively, and the cumulative variance was 76.65% (Table 8). Similar to the first PC at Lujiaowan, Pb and As had relatively higher loadings on PC1, and Zn and Cu had relatively higher loadings on PC2. In general, Pb in unpolluted soil mainly originates from parent material and rock weathering [45]. Whereas, Pb pollution in soil is mainly related to human activities, such as dry and wet deposition of atmospheric Pb, discharge of Pb containing wastewater, and leaching and dialysis of Pb containing waste residue [46]. In agricultural production activities, irrigation wastewater containing Pb also leads to soil Pb pollution. The concentration of As in world soil ranges from 0.1 mg·kg^-1^ to 58.06 mg·kg^-1^, with a median of 6.0 mg·kg^-1^ [47]. The anthropogenic sources of As include industrial sources (As often exists in different heavy metal mines in the form of accompanying elements; thus, the mining and smelting of these heavy metal mines may cause As pollution in the soil around the mining area), agricultural sources (many As-containing compounds are often used as feed additives in the breeding industry; through the reuse of animal excreta, these As compounds and their metabolites are released into farmland), and other sources (some domestic sewage, wastes, and medical drugs often contain As, and the random disposal of these substances increases the risk of As accumulation) [37]. Therefore, we speculate that human disturbances are the primary origin of heavy metal accumulation in our study area. In specific, the waste paper and plastic products discarding and automobile exhaust emissions considerably contributed to the high heavy metal concentrations in our study area. The concentrations of soil Cu and Zn in our study area were higher than the background value. In addition, these two metals had different origins, indicating that Cu and Zn could be complex origin pollutants, similar results were also reported elsewhere [48].

**Table 8:** The principal component analysis results of heavy metal.

Study area	PC	Pb	As	Zn	Cu	Mn	Variance	Cumulative variance
Lujiaowan	PC1	0.28	0.32	-0.25	-0.01	-0.30	59.12	59.12
	PC2	-0.08	-0.04	-0.41	0.74	0.21	26.40	85.52
Juhuatai	PC1	0.37	0.42	0.08	-0.03	0.40	45.13	45.13
	PC2	0.13	-0.42	0.42	0.48	0.08	31.52	76.65

## 3.5 Geoaccumulation and potential ecological risk

**Fig 5:**
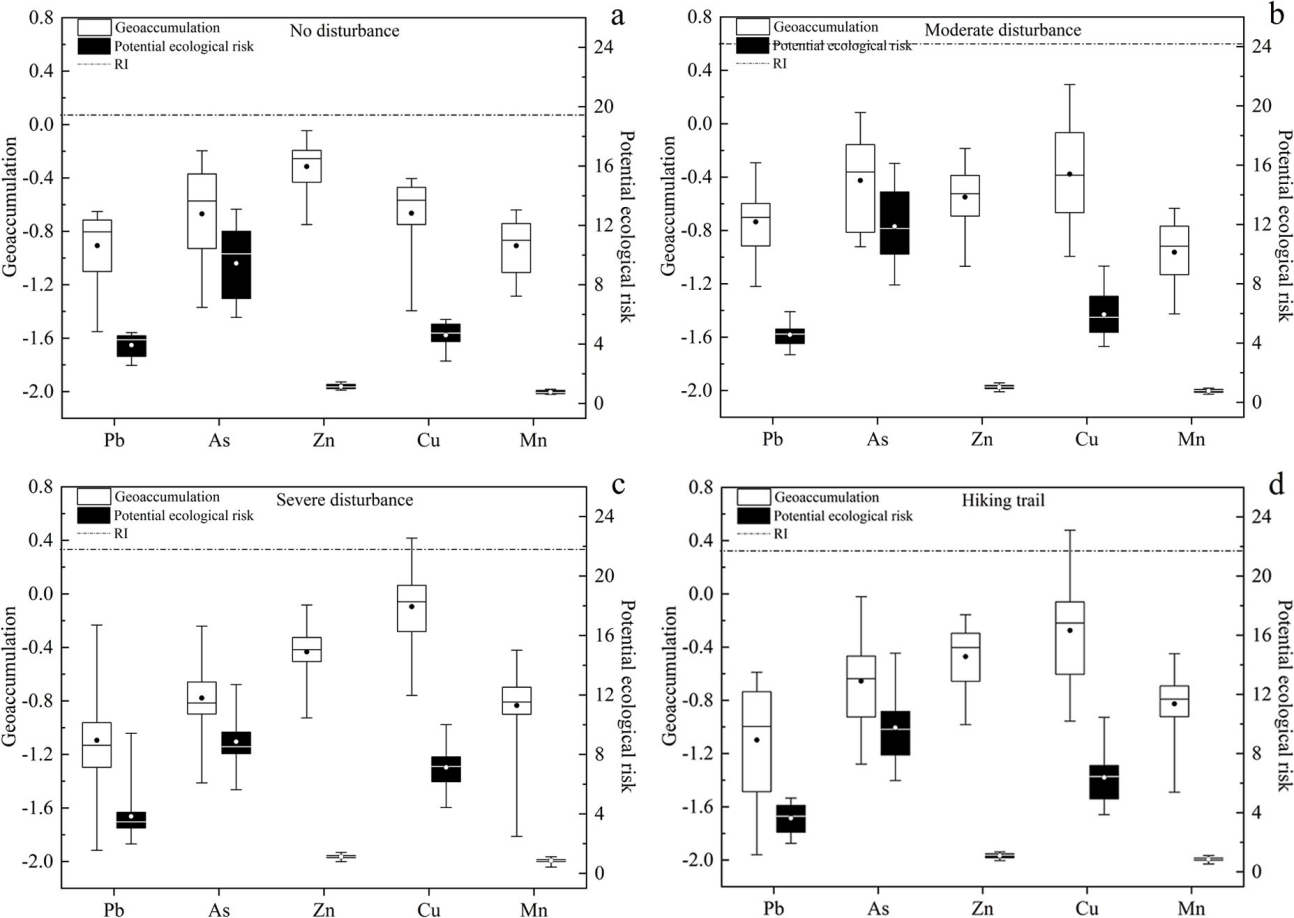
Geoaccumulation and potential ecological risk of soil heavy metal in Lujiaowan Scenic Area.

**Fig 6:**
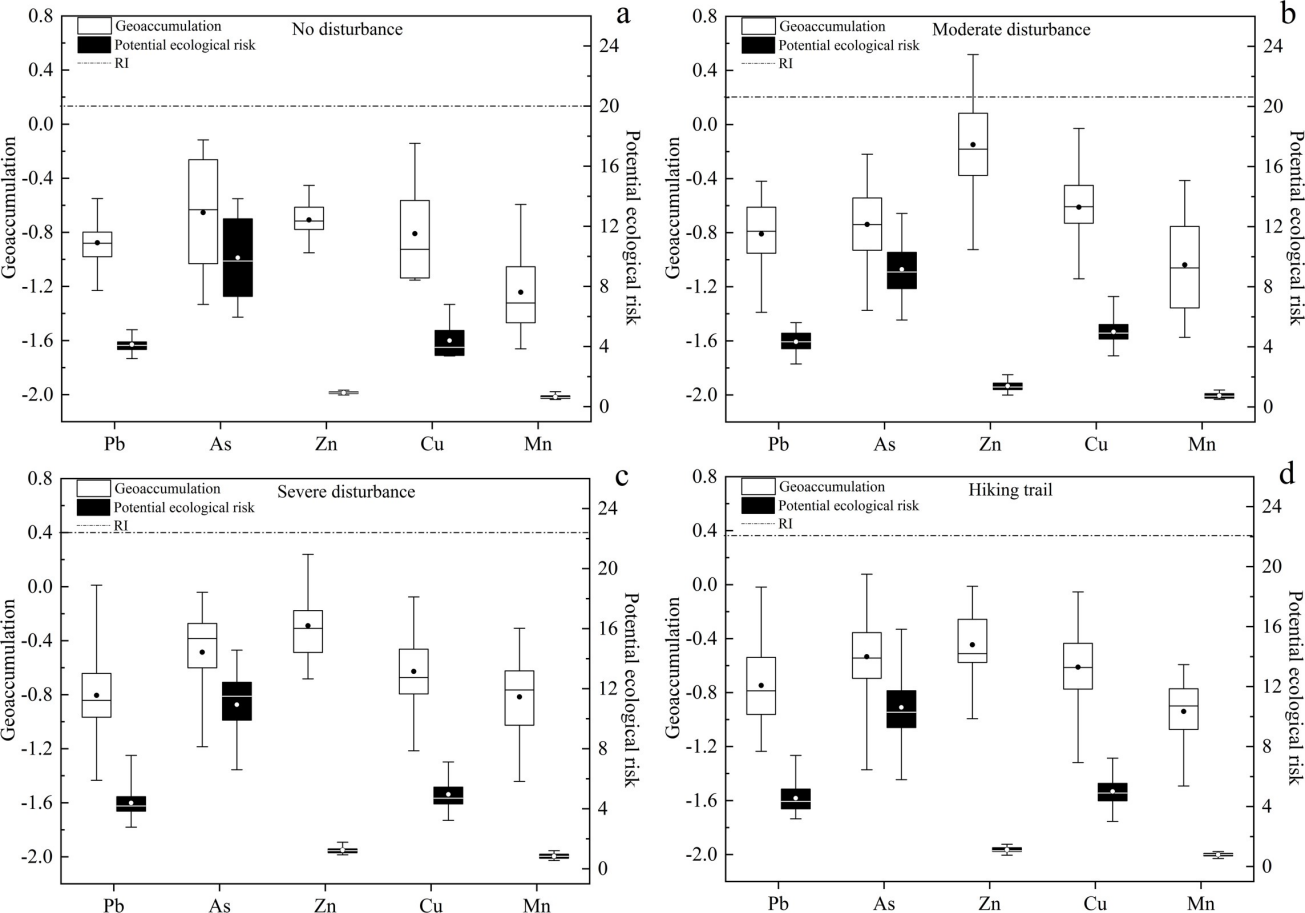
Geoaccumulation and potential ecological risk of heavy metal in Juhuatai Scenic Area.

The average I_geo_ of the five heavy metals in soil under different disturbance conditions in the study area was less than 0 (Figs 5 and 6), indicating that the pollution degree of the five heavy metals was low compared with the background value of soil heavy metals in Xinjiang. The potential ecological risks of five metals were less than 40, which could be classified to low risk. The order of average single-factor pollution index was As > Cu > Pb > Zn > Mn, which demonstrated that the ecological risk of As was the highest. The *RI* of the five soil heavy metals were less than 150, which can be classified to low risk. The *RI* values of the artificially disturbed area were significantly higher than those of the undisturbed areas, for example, the *RI* in Juhuatai area showed severe disturbance > moderate disturbance > no disturbance, indicating that the potential ecological risk level in the study area was closely related to the impact of human activities. More generally, the contamination of soil heavy metal in Xinjiang is not serious. For example, pollution and ecological risk assessment of heavy metals in farmland soils in Yanqi county of Xinjiang province found that compared to the classification standard, Cd, Cr, Cu, As, Mn, and Ni showed no-pollution level [49]. An assessment of heavy metal pollution of agricultural land in the Southern Margin of Tarim Basin in Xinjiang revealed that the soil was only slightly contaminated with Hg and Cd [50].

The order of average concentration of heavy metals in the soil of the study area was Mn > Zn > Cu > Pb > As, and the average concentrations of Zn, Cu and As exceeded the background value of Xinjiang soil. Existing research reported that Cu has good corrosion resistance, thus, it is often used as the material of automobile cooling system and the source of coatings for scenic buildings, this might induced the relatively higher contamination level of Cu in our study area. Zn is also often as the material of automobile lubricants [51, 52], also contributed to the relatively its higher concentration in soil. In addition, transportation is the main influencing factor of Cu and Zn in soils, as mentioned previously [53]. Under different disturbance levels, the heavy metal concentrations in soil were different, as revealed by present study. This result can be ascribed to the fact that areas with strong disturbance have relatively more external heavy metal input to the soil and thus suffer a relatively heavy degree of pollution, similar results has been reported previously [31].

## 4. Conclusions

1. The concentrations of Pb, As, Zn, Cu and Mn in soils of our study area were lower than the national II standard for soil environmental quality. In the 0-10cm soil layer, the average concentrations of Zn (76.19 mg·kg^-1^, 87.33 mg·kg^-1^), Cu (31.93 mg·kg^-1^, 27.84 mg·kg^-1^), and As (11.48 mg·kg^-1^, 11.27 mg·kg^-1^) in the soil of Lujiaowan and Juhuatai scenic spots were higher than the background values of soils in Xinjiang. To the extent of light pollution.
2. Due to both natural factors and human activities, the average content of heavy metals in the five soils of the study area showed a certain regularity. The intensity of disturbance was positively correlated with the concentration of heavy metals.
3. The sources of five heavy metals were complex. Pb and As in the soil of the study area mainly come from human activities such as domestic waste, transportation, industrial activities and agricultural fertilization; Cu and Zn are brought by automobile cooling system materials, scenic architectural coatings and automobile lubricants, respectively.
4. The geoaccumulation index values indicated that the study area can be categorized as “uncontaminated” on the basis of the background value in Xinjiang. The order of potential ecological risk was As > Cu > Pb > Zn > Mn, suggesting low risk level.

## Supporting information

S1 Data (XLSX)
